# Altered Frontal Lateralization Underlies the Category Fluency Deficits in Older Adults with Mild Cognitive Impairment: A Near-Infrared Spectroscopy Study

**DOI:** 10.3389/fnagi.2016.00059

**Published:** 2016-03-29

**Authors:** Michael K. Yeung, Sophia L. Sze, Jean Woo, Timothy Kwok, David H. K. Shum, Ruby Yu, Agnes S. Chan

**Affiliations:** ^1^Department of Psychology, The Chinese University of Hong KongNew Territories, Hong Kong SAR, China; ^2^Chanwuyi Research Center for Neuropsychological Well-Being, The Chinese University of Hong KongNew Territories, Hong Kong SAR, China; ^3^Department of Medicine and Therapeutics, The Chinese University of Hong KongNew Territories, Hong Kong SAR, China; ^4^School of Public Health, The Chinese University of Hong KongNew Territories, Hong Kong SAR, China; ^5^Menzies Health Institute Queensland and School of Applied Psychology, Griffith UniversityGold Coast, QLD, Australia

**Keywords:** mild cognitive impairment (MCI), verbal fluency, category fluency, prefrontal cortex, near-infrared spectroscopy (NIRS), lateralization

## Abstract

Individuals with mild cognitive impairment (MCI) have been consistently found to have category fluency deficits. However, little is known about the neural basis of these deficits. A diversity of neuroimaging studies has revealed left-lateralized prefrontal activations due to verbal processing and control functions during the performance of category fluency tasks. Given the reports of structural and functional abnormalities in the prefrontal cortices in individuals with MCI, it is conceivable that these individuals would also exhibit altered prefrontal activation patterns during a category fluency task. The present study aimed to investigate the prefrontal dynamics during the category fluency task in older adults with MCI by using near-infrared spectroscopy (NIRS). Twenty-six older adults with MCI were compared with 26 older adults with normal cognition (NC) who were matched in age, gender, handedness, and educational level. All participants performed a category fluency task while the prefrontal dynamics were recorded. The results showed that the MCI group generated fewer unique words, made fewer switches between subcategories, and generated fewer new subcategories than did the NC group. Importantly, the NIRS results showed that the NC group exhibited a left lateralization of frontal activations during the category fluency task, while the MCI group did not exhibit such a lateralization. Furthermore, there was a significant positive correlation between the category fluency performance and the extent of lateralization, suggesting that the category fluency deficits in the MCI group could be related to frontal dysfunction. That is, the rightward shift of frontal activations in the MCI group may reflect the presence of cortical reorganization in which the contralateral regions (i.e., the right hemisphere) are recruited to take over the function that is declining in the specialized regions (i.e., the left hemisphere). Our lateralization finding may serve as an objective neural marker for distinguishing between normal aging and MCI. Our study highlights that an alteration of neural functioning is already present at the prodromal stage of dementia.

## Introduction

Mild cognitive impairment (MCI) is considered to be a transitional stage between healthy aging and dementia. Individuals with MCI are concerned about a cognitive decline and exhibit objective cognitive impairment but have essentially normal activities of daily living and are not demented (Albert et al., 2011; Petersen et al., 2014). The MCI construct is heterogeneous. Based on the presence of memory deficits, MCI can be broadly classified into the amnestic and non-amnestic subtypes (Petersen et al., 2014), which have been found to be associated with different etiologies (Yaffe et al., 2006). With regard to neuroanatomy, the amnestic subtype has major disturbances in medial temporal lobe structures, while non-amnestic subtype has major disturbances in other brain structures, such as the frontal cortex when executive dysfunction is evident (e.g., Chao et al., 2009; Pa et al., 2009). The conversion rate of MCI to dementia has been estimated to be approximately 10–20% per annum (Petersen et al., 1999; Amieva et al., 2004; Venneri et al., 2011; Wang et al., 2011). Because early detection of individuals at the prodromal stage of dementia offers the best potential for intervention (Ernst et al., 1997), it is important to identify objective markers, such as neuropsychological and neurophysiological characteristics that can reliably distinguish MCI from normal aging.

The category fluency test is a commonly used neuropsychological test that requires individuals to generate as many words as possible that belong to a semantic category (e.g., animal names) within a given time interval. This task requires the initiation of a specific verbal behavior, a controlled and strategic search of specific verbal information, and self-monitoring to avoid intrusions and repetitions (Bertola et al., 2014). The test has been found to be a significant predictor of future cognitive decline in both the cognitively normal (e.g., Tierney et al., 2005) and MCI populations (e.g., Molinuevo et al., 2011; Venneri et al., 2011). Numerous empirical studies have consistently found that individuals with MCI, irrespective of their subtypes, exhibited category fluency impairment (Murphy et al., 2006; Nutter-Upham et al., 2008; Brandt and Manning, 2009; Libon et al., 2010; Wang et al., 2011; Price et al., 2012; Weakley et al., 2013; Bertola et al., 2014; Rinehardt et al., 2014; Vogel et al., 2014). This impairment has been mainly attributed to their deficits in generating new subcategories or switching between subcategories (Raoux et al., 2008; Price et al., 2012; Weakley et al., 2013; Bertola et al., 2014), although impairment in clustering (Price et al., 2012) may also underlie their fluency deficits. In sum, these findings provide strong evidence for the presence of a category fluency deficit in individuals with MCI.

Category fluency has been investigated extensively using neuroimaging techniques, such as positron emission topography (PET; e.g., Gourovitch et al., 2000), functional magnetic resonance imaging (fMRI: e.g., Weiss et al., 2003; Birn et al., 2010; Meinzer et al., 2013; for a recent meta-analysis, see Wagner et al., 2014), near-infrared spectroscopy (NIRS; Schecklmann et al., 2007; Tupak et al., 2012; Dan et al., 2013; Heinzel et al., 2013, 2015; Liu et al., 2014; Marumo et al., 2014; Holper et al., 2015), and functional transcranial Doppler sonography (fTCD; Gutierrez-Sigut et al., 2015). These studies have consistently found that the prefrontal cortices activate during the performance of category fluency tasks in healthy individuals, reflecting the control functions exerted by the (left inferior) prefrontal cortex in semantic tasks (Thompson-Schill et al., 1997). In addition, a majority has reported a left lateralization of these frontal activations (e.g., Gourovitch et al., 2000; Weiss et al., 2003; Birn et al., 2010; Tupak et al., 2012; Gutierrez-Sigut et al., 2015; Heinzel et al., 2015), most likely reflecting the general dominance of the left hemisphere in verbal functioning (Hellige, 1993). In addition, the absence of a left frontal lateralization (i.e., bilateral or right-dominant activation patterns) during verbal tasks has been found in some disorders and diseases that exhibit verbal or category fluency impairment, including Alzheimer’s dementia (Fallgatter et al., 1997), schizophrenia (Sommer et al., 2001; Weiss et al., 2004), and developmental dysphasia (de Guibert et al., 2011). A focal left-brain injury has also been shown to result in category fluency deficits in addition to a selective reduction of left frontal lateralization during performance (Raja Beharelle et al., 2010). In sum, left-lateralized frontal activations have been observed during category fluency performance, and a reduction or a reversal of lateralization has been documented in individuals with neurocognitive impairment.

Recent neuroimaging studies have revealed frontal abnormality in individuals with MCI. Specifically, some MRI studies have reported a cortical atrophy in the (left) prefrontal cortices in individuals with amnestic or non-amnestic MCI (Pa et al., 2009; Wang et al., 2009; Chang et al., 2010; Ahn et al., 2011; Zhao et al., 2015). In addition, a link between poor semantic fluency performance and cortical atrophy of the lateral prefrontal regions (especially in the left hemisphere) has also been reported in these individuals (Ahn et al., 2011; Eastman et al., 2013; Clark et al., 2014). Furthermore, some fMRI studies have shown that these individuals exhibit abnormal frontal activations while performing various cognitive tasks, including episodic memory retrieval (Heun et al., 2007), semantic memory (Woodard et al., 2009), divided attention (Dannhauser et al., 2005), interference control (Kaufmann et al., 2008; Van Dam et al., 2013), and working memory (Alichniewicz et al., 2012; Papma et al., 2013; see Li et al., 2015, for a recent meta-analysis of fMRI studies in MCI). In summary, structural and functional abnormalities in the prefrontal cortices have been reported in the MCI population, regardless of the subtype.

Given that category fluency seems to be mediated by the frontal lobe and that individuals with MCI have shown frontal dysfunction, it is reasonable to hypothesize that individuals with MCI will demonstrate abnormal frontal activations during category fluency performance. In addition, a reduction in left-lateralized frontal activations has been shown as an indicator of frontal dysfunction during the fluency task. Thus, it is anticipated that individuals with MCI will demonstrate a reduced lateralization of frontal activations during the category fluency task. As a further investigation, we would compare the amnestic and non-amnestic MCI subtypes given that the MCI construct is heterogeneous (Petersen et al., 2014).

The NIRS is an optical imaging method that uses light in the near-infrared spectrum (700–1000 nm) to non-invasively monitor the hemodynamic responses evoked by brain activity (Villringer and Chance, 1997). It measures the quantitative changes of the oxygenated hemoglobin (oxy-Hb) and deoxygenated hemoglobin (deoxy-Hb) concentration in the cerebral blood. These NIRS signals have been shown to correlate with the blood oxygenation level dependent (BOLD) signals as measured by the fMRI (Strangman et al., 2002; Cui et al., 2011; Sato et al., 2013). The NIRS has been extensively used to study the prefrontal brain dynamics during the category fluency task in healthy adults (Hori et al., 2008; Kahlaoui et al., 2012; Tupak et al., 2012; Dan et al., 2013; Heinzel et al., 2013, 2015) and those with psychiatric disorders or neurological diseases, including schizophrenia (Kubota et al., 2005; Ehlis et al., 2007; Marumo et al., 2014), major depression disorder (Liu et al., 2014), and Alzheimer’s Disease (Fallgatter et al., 1997). Previous NIRS studies with healthy adults have consistently found [oxy-Hb] increases in the prefrontal cortices (predominantly lateral regions) during the category fluency task (Schecklmann et al., 2007; Tupak et al., 2012; Dan et al., 2013; Heinzel et al., 2013, 2015; Liu et al., 2014; Marumo et al., 2014; Holper et al., 2015). In addition, many of these have reported a left lateralization of [oxy-Hb] increases at the lateral prefrontal regions (Fallgatter et al., 1997; Tupak et al., 2012; Dan et al., 2013; Heinzel et al., 2013, 2015) and thus are largely consistent with the fMRI literature (fMRI: e.g., Weiss et al., 2003; Meinzer et al., 2013; for review, see Wagner et al., 2014; fTCD: Gutierrez-Sigut et al., 2015). Therefore, accumulating evidence has supported the use of NIRS in studying the prefrontal dynamics during the category fluency task, in which left-lateralized frontal activations (i.e., [oxy-Hb] increases) could be detected (e.g., Tupak et al., 2012; Dan et al., 2013; Heinzel et al., 2015). Thus, the present study utilized NIRS to study the frontal activations associated with category fluency in individuals with MCI.

## Materials and Methods

### Participants

The original sample included 69 older adults who were aged between 60 and 91 years. The participants were recruited through advertisements in the community and from the health and social centers in the New Territories East regions in Hong Kong. They were invited to engage in an intervention program on memory. As a pre-intervention assessment, all of the participants underwent a comprehensive neuropsychological assessment and a NIRS recording session. Exclusion criteria were as follows: (1) a score higher than 7 on the short form of the Chinese Geriatric Depression Scale (CGDS-SF; Lee et al., 1993); (2) a score higher than 15 (i.e., in the moderate or severe range) on the Beck Anxiety Inventory (BAI; Beck et al., 1988); (3) a history of head injury or neurological/psychiatric disorders; and (4) signs of dementia (e.g., impaired on all neuropsychological measures). Following the criteria proposed by Petersen et al. (2014) and the National Institute on Aging-Alzheimer’s Association workgroups (Albert et al., 2011), participants were considered to have MCI if they met the following criteria: (1) a concern regarding a change in cognition; (2) an objective cognitive impairment; (3) a preservation of independence in functional abilities; and (4) not being demented. The objective cognitive impairment was operationalized in accordance with the comprehensive neuropsychological criteria proposed by Jak et al. (2009), in which participants were considered to have MCI if they scored more than 1 SD below the age- (and education-) corrected normative mean on at least two measures within at least one cognitive domain (i.e., memory, speed/executive function, language, and visuospatial function; see Table 1). The use of these criteria, instead of a single impaired score, has been shown to reduce false positive errors in detecting MCI (Clark et al., 2013; Bondi et al., 2014). On the other hand, participants were considered to be cognitively normal if, at most, performance on one measure within one or two cognitive domains fell more than 1 SD below the age- (and education-) corrected norms (Jak et al., 2009).

**Table 1:** **Neuropsychological measures used in the present study**.

Memory	Speed/executive function	Language	Visuospatial function
HKLLT: total learning	STT-A: time	CF (animals): total unique words in 60 s	WMS-VR: copy
HKLLT: delayed recall	STT-B: time	CF (transportation): total unique words in 60 s	RCFT: copy score
WMS-VR: immediate recall	RCFT: copy organization	BNT: spontaneous naming	
WMS-VR: delayed recall

*Note: BNT, Boston Naming Test; CF, Category Fluency; HKLLT, Hong Kong List Learning Test; RCFT, Rey-Osterrieth Complex Figure Test; STT, Shape Trail Test; WMS-VR, Wechsler Memory Scale-Visual Reproduction. An objective cognitive impairment for mild cognitive impairment (MCI) was operationalized by the scoring of more than 1 SD below the age- (and education-) corrected normative mean on at least two measures within at least one cognitive domain (i.e., memory, speed/executive function, language, and visuospatial function)*.

The final sample consisted of 26 older adults with MCI and 26 older adults with normal cognition (NC) after excluding participants who were unable to complete the tests in the NIRS recording session and matching the two groups on mean age and educational level, gender, and handedness (*p* > 0.10). According to Petersen et al. (2014), 10 older adults with MCI belonged to the amnestic subtype, and 16 belonged to the non-amnestic subtype. Because there were no significant differences between the two MCI groups except for memory measures, *p*s > 0.05, and because the sample size of each group was small, we mainly present the results with the combined MCI group. The demographic and neuropsychological characteristics of the NC and MCI groups are shown in Table 2. All of the participants provided written informed consent prior to the study. The study was approved by the Joint Chinese University of Hong Kong-New Territories East Cluster (CUHK-NTEC) Clinical Research Ethics Review Committee.

**Table 2:** **Demographic and neuropsychological characteristics of the mild cognitive impairment (MCI) and normal cognition (NC) groups**.

	Group	
	MCI (*n* = 26)	NC (*n* = 26)	*t/Λ*	*p*
Age (yrs)	69.07 (6.20)	68.87 (6.08)	0.12	0.91
Gender (M/F)	6/20	7/19	0.10	0.75
Handedness (R/L)	25/1	25/1	0.00	1.00
Education (yrs)	7.94 (4.41)	9.58 (3.26)	1.52	0.14
**Memory**
HKLLT: total learning	20.92 (6.42)	24.00 (5.50)	1.86	0.069^†^
HKLLT: delayed recall	6.35 (2.87)	8.08 (2.95)	2.14	0.037*
WMS-VR: immediate recall	61.04 (15.96)	71.62 (10.16)	2.85	0.006**
WMS-VR: delayed recall	36.62 (17.41)	51.15 (21.89)	2.65	0.011*
**Speed/executive functions**
STT-A: time (s)	69.19 (21.99)	54.54 (16.03)	2.75	0.008**
STT-B: time (s)	216.27 (108.75)	140.00 (49.80)	3.25	0.003**
RCFT: copy organization	5.50 (2.29)	6.85 (1.26)	2.63	0.012*
**Language**
CF (animals): unique words	14.04 (4.64)	18.00 (5.03)	2.95	0.005**
CF (transportation): unique words	10.15 (2.96)	11.92 (2.83)	2.2	0.032*
BNT: spontaneous naming	17.15 (4.05)	21.04 (2.27)	4.27	<0.001***
**Visuospatial functions**
WMS-VR: copy	91.35 (5.38)	95.38 (4.36)	2.97	0.005**
RCFT: copy score	28.54 (7.83)	32.75 (2.46)	2.62	0.014*
**Global functioning level**
CDRS: adjusted total score	149.34 (6.67)	153.44 (6.25)	2.27	0.028*
**Questionnaires**
ADL-PI-Self: daily functioning	38.67 (4.94)	38.00 (4.13)	0.51	0.61
ADL-PI-Self: physical functioning	4.88 (0.34)	4.88 (0.34)	0.00	1.00
BAI	2.73 (2.99)	4.15 (3.60)	1.55	0.13
CGDS-SF	2.12 (1.93)	2.62 (2.08)	0.90	0.37

Note: ADL-PI-Self, Self-report version of the Activities of Daily Living-Prevention Instrument; BAI, Beck Anxiety Inventory; BNT, Boston Naming Test; CDRS, Chinese Version of the Mattis Dementia Rating Scale; CF, Category Fluency; CGDS, Chinese Geriatric Depression Scale-Short Form; HKLLT, Hong Kong List Learning Test; RCFT, Rey-Osterrieth Complex Figure Test; STT, Shape Trail Test; WMS-VR, Wechsler Memory Scale-Visual Reproduction. There were four missing values for the ADL-PI-Self (MCI: n = 2; HC: n = 2), and 1 for the CDRS (NC: n = 1). Standard deviations are in parentheses. ^†^p < 0.10; *p < 0.05; **p < 0.01; ***p < 0.001.

### Measures and Procedure

For the neuropsychological assessment, all of the participants underwent a neuropsychological protocol that included the following tests: the Hong Kong List Learning Test (HKLLT; Chan and Kwok, 2006) and the Visual Reproduction of the Wechsler Memory Scale (WMS-VR; Wechsler, 2005) as the memory tests; The Shape Trail Test (STT; Zhao et al., 2013) and the Rey-Osterrieth Complex Figure Test (RCFT; Bernstein and Waber, 1996) as the tests for speed/executive function; the Boston Naming Test (BNT; Cheung et al., 2004) as the test for language; and the WMS-VR and RCFT as the tests for visuospatial function. In addition, the Chinese version of the Mattis Dementia Rating Scale (CDRS; Chan et al., 2001) was also administered to estimate the global functioning level. The short form of the Chinese Geriatric Depression Scale (CGDS-SF) and the Beck Anxiety Inventory (BAI) were used to assess depression and anxiety levels. The self-report version of the Activities of Daily Living-Instrument Prevention (ADL-PI-Self; Galasko et al., 2006) was used to assess daily and physical functioning levels. The assessment was administered by trained research assistants.

The design of the category fluency task was adapted from previous NIRS studies of verbal fluency (e.g., Tupak et al., 2012; Liu et al., 2014) in which the task blocks were interleaved with control blocks (i.e., a blocked design). For the task blocks, the participants had to generate words that belong to a particular semantic category, of which the category word was shown at the center of a computer screen. Animal and means of transportation were employed as the categories for word generation in a fixed order in accordance with a previous Chinese normative study (Chan and Poon, 1999) that would allow the conversion of *Z* scores for MCI classification. For the control blocks, the participants had to slowly repeat “1, 2, 3, 4”, of which the phrase was shown at the center of the screen, to control for the effect of overt verbal production.

Before the experiment began, participants were first briefed about the task instructions and experimental design, and flower words were used as the category example. They were asked to generate as many words as possible during the task blocks. They were also asked to open their eyes and to avoid making any movements throughout the experiment. The experiment began with a 30-s control period, followed immediately by a 60-s task period (i.e., animal words), a 60-s control period, another 60-s task period (i.e., transportation words), and ended with another 60-s control period. The total measurement period was 270 s. The stimuli were presented using the E-Prime 1.2 Software (Psychology Software Tools, Pittsburgh, PA, USA). The NIRS session was administered by a doctorate student and a trained research assistant.

### NIRS Measurement

**Figure 1:**
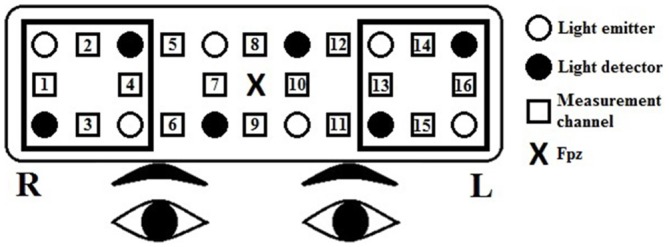
**Arrangement of the near-infrared spectroscopy (NIRS) channels.** Channels 13–16 represent the left prefrontal region, and channels 1–4 represent the right prefrontal region. The distance between pairs of emitter and detector probes was 3 cm. In accordance to the international 10/20 system, the center of the probe matrix was placed on Fpz, and the probes at the bottom left and right corners were placed around F7 and F8, respectively.

The relative [oxy-Hb] and [deoxy-Hb] changes were recorded using a 16-channel OEG-SpO2 system (Spectratech Inc., Tokyo, Japan). The machine uses two wavelengths of near-infrared light, 770 and 840 nm, and calculates the amount of absorbed near-infrared light based on the modified Beer-Lambert Law (Delpy et al., 1988). Six emission and six detector probes were arranged in a 2 rows × 6 columns matrix on the participant’s forehead (see Figure 1). The distance between pairs of emitter and detector probes was 3 cm, thus measuring the [oxy-Hb] changes at a depth of 2–3 cm below the scalp (Toronov et al., 2001). The NIRS data were collected at 16 measurement points that were located in between each pair of emitter and detector probes. In accordance to the international 10/20 system (Jasper, 1958), the center of the probe matrix was placed on Fpz, and the probes at the bottom left and right corners were placed around F7 and F8, respectively. The sampling rate was 12.21 Hz. Because the [oxy-Hb] signals have been shown to have a better signal-to-noise ratio and demonstrate a stronger relationship with the BOLD signal as measured by the fMRI or with the cerebral blood flow compared to the [deoxy-Hb] signals (Strangman et al., 2002; Cui et al., 2011), we investigated only the [oxy-Hb] data in the present study.

### Data Analysis

For the non-categorical demographic and neuropsychological data, the MCI and NC groups were compared using independent sample *t*-tests. For the categorical variables (i.e., gender and handedness), the groups were compared using the Likelihood Ratio tests. We also further investigated the cognitive processes (i.e., clustering and switching) underlying the animal fluency task as proposed by Troyer (2000). Clustering is an automatic process that involves the generation of words within semantic subcategories and reflects semantic associations, whereas switching is an effortful executive process that reflects flexible thinking. The scoring procedure and listing can be found in Troyer. In brief, the mean cluster size referred to the mean number of successively generated words that belong to a same subcategory, such as African animals, canines, etc., including intruded and repeated words. Clustering starts with a second successive word. For example, two related words constitute a cluster size of 1, or three related words constitute a cluster size of 2. The number of switches refers to the number of switches between subcategories. New subcategory generation, which measures the ability to access semantic subcategories, was also investigated (Price et al., 2012; Bertola et al., 2014).

For the [oxy-Hb] data, a low-pass filter at 0.10 Hz and a slope of 60 dB/octave was first applied to remove short-term motion and cardiac artifacts. Then, we performed a first-order linear fitting, based on the mean values of the pre- and post-task baseline periods, defined by the 10 s preceding the 60-s task block and the last 10 s of the following control block, respectively, to exclude the task-unrelated changes during the two 60-s task blocks. The baseline-corrected data were then averaged across time points for each fluency condition, channel, and participant. The baseline correction and averaging were performed using Matlab^®^ R2014a (The MathWorks, Natick, MA, USA). Because both fMRI (Wagner et al., 2014) and NIRS (e.g., Tupak et al., 2012) studies have shown the lateral prefrontal cortex as the main site of activation during the category fluency task, we conducted a region-of-interest (ROI) analysis: channels 13–16 represent the left prefrontal region, and channels 1–4 represent the right (Makizako et al., 2013).

To quantify the lateralization of frontal activations (i.e., [oxy-Hb] increases), we adopted a modified version of the laterality index that is formulated as (A_L_−A_R_)/(|A_L_| + |A_R_|; Seghier, 2008). A_L_ is the mean [oxy-Hb] changes on the left side, and A_R_ is the mean [oxy-Hb] changes on the right. The absolute value sign in the formula was suggested by Seghier (2008) to take into account negative values. The laterality index is a ratio measure that reflects differences in the activation level between the two hemispheres in proportion to the overall activation level on both sides, ranging from −1 to 1. A positive value indicates a left lateralization, whereas a negative value indicates a right lateralization. The laterality index takes into account individuals’ overall activation level and allows the comparison of lateralization between groups without being confounded by group differences in activation level. The laterality index has been used in some NIRS studies to investigate lateralization (Chaudhary et al., 2011; Kahlaoui et al., 2012). The laterality index was first calculated for each homologous channel pair (e.g., ch1–ch16, ch2–ch14, ch3–ch15, etc.), fluency condition, and participant. Then, we averaged the laterality index across the lateral channel pairs (i.e., ch1–ch16, ch2–ch14, ch3–ch15, and ch4–ch13) and fluency conditions for each participant prior to the statistical analyses. We then tested the mean laterality index against zero using one-sample *t*-tests to investigate lateralization within each group before comparing the MCI and NC groups with an independent sample *t*-test. A similar statistical procedure was repeated for the magnitude of [oxy-Hb] changes to investigate the frontal activation level during the category fluency task within and between the groups.

To further investigate the relationship between the NIRS data (i.e., the laterality index and mean [oxy-Hb] changes) and the behavioral performance of category fluency (i.e., total number of words generated in 60 s), Pearson’s correlations (two-tailed) were also calculated. All of the statistical analyses were performed using SPSS 22.0 Software (IBM Corporation, Armonk, NY, USA). The significance level was set at 0.05 for all tests.

## Results

### Demographic and Neuropsychological Characteristics

The demographic and neuropsychological characteristics of the MCI and NC groups are presented in Table 2. For the demographic variables, the two groups did not differ significantly in age, education level, gender, or handedness, all *p*s > 0.10. For the neuropsychological variables, the MCI group performed significantly worse than the NC group on almost all of the measures (*p*s < 0.05). For memory, the MCI group tended to learn significantly fewer words and recalled significantly fewer words on the HKLLT, and they recalled fewer figures immediately after presentation and after a delay on the WMS-VR than did the NC group. For speed/executive functions, the MCI group was significantly slower on both conditions of the SST and had significantly poorer organization in copying on the RCFT than did the NC group. For language, the MCI group generated significantly fewer unique animal words, *t*_(50)_ = 2.95, *p* = 0.005, *d* = 0.82, and transportation words, *t*_(50)_ = 2.20, *p* = 0.032, *d* = 0.61, on the category test during the NIRS recording session and had significantly poorer spontaneous naming ability on the BNT than did the NC group. For visuospatial function, the MCI group had significantly poorer accuracy in copying figures both on the WMS-VR and RCFT. Furthermore, the MCI group had a significantly lower CDRS score than did the NC group. However, the MCI and NC groups did not differ significantly in daily and physical functioning levels or depression and anxiety levels (*p*s > 0.05).

### Further Analyses for the Category Fluency Task Performance

Previous studies have found that the category fluency deficits in individuals with MCI seemed to be mainly attributed to their deficits in generating new subcategories or switching between subcategories (e.g., Bertola et al., 2014), although deficits in creating large clusters might also be a contributing factor (Price et al., 2012). Therefore, we analyzed the underlying cognitive processes, clustering and switching as proposed by Troyer (2000) and new subcategory generation as used by Bertola et al. (2014) during the animal fluency task. The results of the analyses are presented in Table 3. The independent sample *t*-test results showed that the MCI group made significantly fewer switches between subcategories, *t*_(50)_ = 3.08, *p* = 0.003, *d* = 0.86, and generated significantly fewer new subcategories than did the NC group, *t*_(50)_ = 3.93, *p* < 0.001, *d* = 1.09 (see Table 3). The mean cluster size did not significantly differ between the groups, *t*_(50)_ = 0.04, *p* = 0.97, *d* = 0.01. In summary, the results suggest that the reduced animal word generation in the MCI group seemed to be attributed to their impairment in flexible thinking and strategic search and access of semantic information.

**Table 3:** **Quantitative analysis of the cognitive processes underlying the category fluency tasks in the MCI and NC groups**.

	Group	
	MCI (*n* = 26)	NC (*n* = 26)	*t*	*p*
Mean cluster size	1.06 (0.52)	1.07 (0.64)	0.04	0.97
Number of switches	6.62 (2.50)	9.27 (3.61)	3.08	0.003**
Number of new subcategories	5.15 (1.71)	7.27 (2.15)	3.93	<0.001***

Note: Standard deviations are in parentheses. **p < 0.01; ***p < 0.001.

The reduced word generation in the MCI group might be simply due to the use of impaired category fluency scores (i.e., language impairment) for MCI classification. To investigate the effect of this, we ran the analyses again after excluding the three participants that were classified into the MCI group solely due to language impairment. None of the results significantly changed. Therefore, individuals with MCI that had objective impairment in non-language domains also had poorer category fluency compared to those with NC. In addition, processing speed may confound the category fluency deficits in the MCI group because the STT-A time was significantly correlated with the total number of animal and transportation words, *r*_(50)_ = −0.37, *p* = 0.007. We ran an Analysis of covariance (ANCOVA) with the STT-A time as the covariate, and the MCI group still generated fewer total unique words than did the NC group, *F*_(1,49)_ = 6.41, *p* = 0.015, *η_p_* = 0.12. Thus, processing speed did not seem to be a confounding factor.

### NIRS Analysis

**Figure 2:**
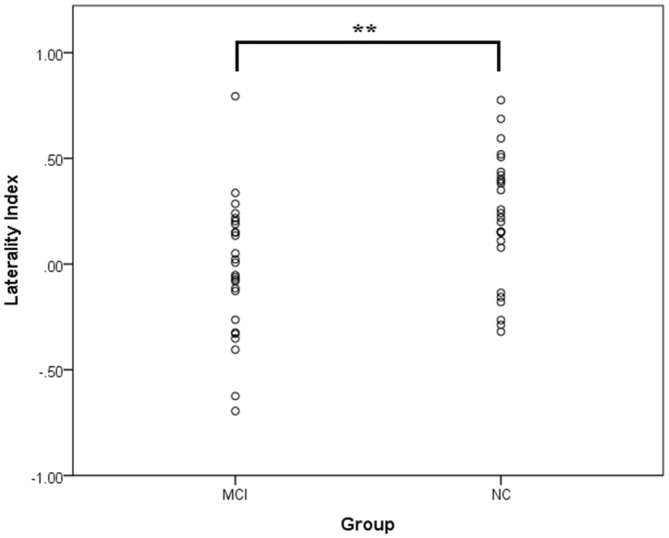
**The laterality index of the mean [oxy-Hb] changes at the lateral prefrontal regions in the mild cognitive impairment (MCI; *n* = 26) and normal cognition (NC; *n* = 26) groups.** A positive value implies a lateralization to the left, whereas a negative value implies a lateralization to the right. No outliers (i.e., two SDs above or below the group mean) were identified. **p < 0.01.

We first investigated the lateralization of oxy-Hb changes using the laterality index, which was averaged over the lateral PFC channel pairs. It should be noted that because the laterality index takes into account the overall activation level on an individual basis, it allows the comparison of the quantity of lateralization between groups without being confounded by group differences in the activation level. A scatter plot of the data is shown in Figure 2. The one-sample *t*-tests showed that the NC group had a significant left lateralization, *t*_(25)_ = 3.68, *p* = 0.001, *d* = 0.72, whereas the MCI group did not, *t*_(25)_ = 0.45, *p* = 0.66, *d* = 0.09. This result suggests a bilateral activation pattern in the MCI group. An independent samples *t*-test showed that the NC group had a significantly larger laterality index than did the MCI group, *t*_(50)_ = 2.86, *p* = 0.006, *d* = 0.79, suggesting that the NC group had a significantly greater left lateralization than did the MCI group. To investigate the confounding effect of the demographic variables, we also performed an ANCOVA with age and education level as the covariates. The group difference in the laterality index remained significant, *F*_(1,48)_ = 7.14, *p* = 0.010, *η*_p_ = 0.13. Thus, the results suggest that compared to the NC group, the MCI group exhibited a reduction and a lack of frontal lateralization during the category fluency task; a finding that was not confounded by group differences in age or education level. The results did not significantly change after excluding the two participants with left handedness, one in each group.

**Figure 3:**
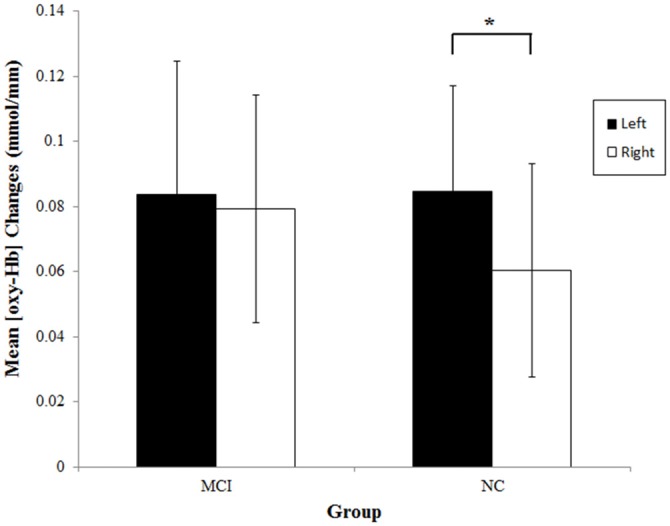
**Mean [oxy-Hb] changes at the left (ch13–16) and right (ch1–4) prefrontal regions during the category fluency tasks in the MCI (*n* = 26) and NC (*n* = 26) groups.** Error bars denote the 95% confidence interval. *p < 0.05.

To examine if the two groups showed significant activations on each side during the category fluency task, we analyzed the mean [oxy-Hb] changes within the left and right prefrontal regions. The data are shown in Figure 3. The results of one-sample *t*-tests showed that the mean [oxy-Hb] on both sides significantly increased in both the NC group [left: *t*_(25)_ = 5.14, *p* < 0.001, *d* = 1.01; right: *t*_(25)_ = 3.61, *p* = 0.001, *d* = 0.71] and the MCI group [left: *t*_(25)_ = 4.02, *p* < 0.001, *d* = 0.79; right: *t*_(25)_ = 4.43, *p* < 0.001, *d* = 0.87], suggesting that the lateral prefrontal cortices were significantly activated in both of the groups. Consistent with the results obtained with the laterality index, the MCI group had similar [oxy-Hb] increases on each side, *t*_(25)_ = 0.39, *p* = 0.70, *d* = 0.05, whereas the NC group exhibited significantly greater [oxy-Hb] increases on the left than the right, *t*_(25)_ = 2.38, *p* = 0.025, *d* = 0.29.

No significant group differences in the mean [oxy-Hb] changes were found [left: *t*_(50)_ = 0.04, *p* = 0.97, *d* = 0.01; right: *t*_(50)_ = 0.77, *p* = 0.45, *d* = 0.21]. None of the results significantly changed after excluding the three participants that were classified into the MCI group solely due to language impairment.

### Relationship Between the NIRS Variables, Category Fluency and Neuropsychological Measures

**Figure 4:**
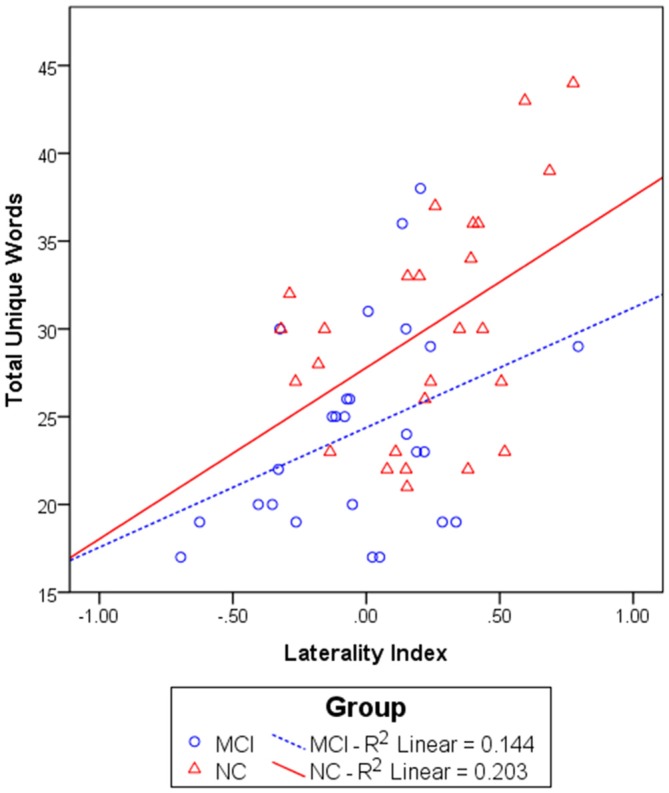
**The relationship between the laterality index and total unique words generated in 60 s in the MCI (*n* = 26) and NC (*n* = 26) groups.** A linear regression line was fit for each group separately.

We first investigated the relationship between the NIRS variables and category fluency performance by calculating Pearson’s correlations. The NIRS variables included the left and right [oxy-Hb] changes and laterality index. The results showed that the laterality index was significantly correlated with the total number of unique animal and transportation words generated for the whole group, *r*_(50)_ = 0.51, *p* < 0.001. To investigate whether this correlation simply reflected a group difference in the laterality index and task performance, correlation analyses were also performed for each group separately. The results showed that the correlation between the laterality index and category fluency performance was marginally significant within the MCI group, *r*_(24)_ = 0.38, *p* = 0.056, and significant within the NC group, *r*_(24)_ = 0.45, *p* = 0.021 (see Figure 4). The results did not significantly change after partialling out the effect of age and education level, both for the whole group, *r*_(48)_ = 0.50, *p* < 0.001, and separately for the MCI group, *r*_(22)_ = 0.43, *p* = 0.038, and the NC group, *r*_(22)_ = 0.43, *p* = 0.035. Thus, the results suggest that a greater left lateralization of frontal activations was associated with a better category fluency performance for each of the groups. Neither the [oxy-Hb] changes in the left nor right prefrontal regions were significantly related to the task performance for the whole group, although the direction of the relationship was as expected [left: *r*_(50)_ = 0.23, *p* = 0.11; right: *r*_(50)_ = −0.04, *p* = 0.79].

Then, to investigate the specificity of the laterality index, we also studied the relationship between the laterality index and other neuropsychological variables. For the neuropsychological variables, one measure was chosen from each cognitive domain to be included for correlational analyses: the delayed recall on the HKLLT for memory, time to completion on trial B of the SST for speed/executive function, spontaneous naming on the BNT for language, and copy score on the RCFT for visuospatial function. The results showed that the laterality index was significantly correlated only with the BNT spontaneous naming score, *r*_(50)_ = 0.34, *p* = 0.013. Thus, the laterality index seems to relate and show some specificity with verbal ability.

### Subgroup Analyses for Amnestic and Non-Amnestic MCI

Because different MCI types may be associated with different etiologies (e.g., Yaffe et al., 2006), we repeated the analyses after dividing the MCI group into the amnestic (aMCI; *n* = 10) and non-amnestic (naMCI; *n* = 16) groups to investigate if MCI subtype moderated the results. The groups were compared using ANOVA, followed by independent sample *t*-tests if the results were significant. The group differences were not significant for each of the demographic variables, *p*s > 0.15, but were significant for almost all of the neuropsychological measures (see Table 4), *p*s < 0.085. As expected, the aMCI group had significantly poorer memory (i.e., poorer total learning and delayed recall on the HKLLT and immediate recall on the WMS-VR) compared to the naMCI and TD groups, *p*s < 0.05. The group differences in memory between the naMCI and NC groups were not significant, *p*s > 0.12. In addition, deficits in speed/executive functions, language, and visuospatial function were more pronounced for the naMCI group and less so for the aMCI group, but the two groups did not significantly differ in these non-memory measures, *p*s > 0.16. For the category fluency task (see Table 5), the group differences were significant for the total number of words generated and the number of switches and new subcategories, *p*s < 0.012. Both MCI groups generated significantly fewer words compared to the NC group (aMCI: *p* = 0.012; naMCI: *p* = 0.011). In addition, compared to the NC group, both MCI groups generated significantly fewer subcategories (aMCI: *p* = 0.045; naMCI: *p* < 0.001) and made fewer switches (aMCI: *p* = 0.089; naMCI: *p* = 0.007). The two MCI groups did not significantly differ, *p*s > 0.20.

**Table 4:** **Demographic, neuropsychological and clinical characteristics of the amnestic (aMCI) and non-amnestic (naMCI) MCI and the NC group**.

	Group		
	aMCI (*n* = 10)	naMCI (*n* = 16)	NC (*n* = 26)	*F/Λ*	*p*
Age (yrs)	68.01 (5.80)	69.74 (6.53)	68.87 (6.08)	0.25	0.78
Gender (M/F)	2/8	4/12	7/19	0.19	0.91
Handedness (R/L)	10/0	15/1	25/1	1.00	0.61
Education (yrs)	6.80 (4.66)	8.66 (4.24)	9.58 (3.26)	1.87	0.16
**Memory**
HKLLT: total learning	16.30 (2.36)^ac^	23.81 (6.50)	24.00 (5.50)	8	0.001**
HKLLT: delayed recall	4.20 (1.48)^ac^	7.69 (2.73)	8.08 (2.95)	7.99	0.001**
WMS-VR: immediate recall	50.40 (16.37)^ac^	67.69 (11.87)	71.62 (10.16)	11.35	<0.001***
WMS-VR: delayed recall	28.60 (20.03)^a^	41.63 (13.95)	51.15 (21.89)	5.02	0.010*
**Speed/executive functions**
STT-A: time (s)	61.60 (17.59)	73.94 (23.62)^b^	54.54 (16.03)	5.20	0.009**
STT-B: time (s)	214.60 (93.16)^a^	217.31 (120.42)^b^	140.00 (49.80)	5.18	0.009**
RCFT: copy organization	5.90 (1.97)	5.25 (2.49)^b^	6.85 (1.26)	3.83	0.028*
**Language**
CF (animals): unique words	13.40 (3.34)^a^	14.44 (5.37)^b^	18.00 (5.03)	4.43	0.017*
CF (transportation): unique words	10.60 (3.75)	9.88 (2.45)^b^	11.92 (2.83)	2.59	0.085^†^
BNT: spontaneous naming	16.40 (4.70)^a^	17.63 (3.67)^b^	21.04 (2.27)	9.51	<0.001***
**Visuospatial functions**
WMS-VR: copy	90.60 (3.50)^a^	91.81 (6.35)^b^	95.38 (7.36)	4.55	0.015*
RCFT: copy score	28.40 (6.31)	28.63 (8.85)^b^	32.75 (2.46)	3.36	0.043*
**Global functioning level**					
CDRS: adjusted total score	151.13 (4.10)	148.21 (7.79)^b^	153.44 (6.25)	3.21	0.049*
**Questionnaires**
ADL-PI-Self: daily functioning	37.44 (6.31)	39.40 (3.98)	38.00 (4.13)	0.65	0.53
ADL-PI-Self: physical functioning	4.89 (0.33)	4.87 (0.35)	4.88 (0.34)	0.01	0.99
BAI	1.80 (2.44)	3.31 (3.22)	4.15 (3.60)	1.86	0.17
CGDS-SF	2.00 (2.21)	2.19 (1.80)	2.62 (2.08)	0.42	0.66

Note: ADL-PI-Self, Self-report version of the Activities of Daily Living-Prevention Instrument; BAI, Beck Anxiety Inventory; BNT, Boston Naming Test; CDRS, Chinese Version of the Mattis Dementia Rating Scale; CF, Category Fluency; CGDS, Chinese Geriatric Depression Scale-Short Form; HKLLT, Hong Kong List Learning Test; RCFT, Rey-Osterrieth Complex Figure Test; STT, Shape Trail Test; WMS-VR, Wechsler Memory Scale-Visual Reproduction. There were four missing values on the ADL-PI-Self (aMCI: n = 1; naMCI: n = 1; NC: n = 2), and 1 for the adjusted total score on the CDRS (NC: n = 1). ^a^The aMCI group performed significantly worse than did the NC group, p < 0.05. ^b^The naMCI group performed significantly worse than did the NC group, p < 0.05. ^c^The aMCI group performed significantly worse than did the naMCI group, p < 0.05. ^†^p < 0.10; *p < 0.05; **p < 0.01; ***p < 0.001.

**Table 5:** **Behavioral performance on the category fluency tasks in the amnestic (aMCI) and non-amnestic (naMCI) MCI and NC groups**.

	Group		
	aMCI (*n* = 10)	naMCI (*n* = 16)	NC (*n* = 26)	*F*	*p*
Mean cluster size	0.90 (0.59)	1.16 (0.52)	1.07 (0.64)	0.63	0.54
Number of switches	7.10 (2.42)	6.31 (2.58)^b^	9.27 (3.61)	4.89	0.012*
Number of new subcategories	5.70 (1.64)^a^	4.81 (1.72)^b^	7.27 (2.15)	8.41	0.001**

Note: Standard deviations are in parentheses. ^a^The aMCI group performed significantly worse than did the NC group, p < 0.05. ^b^The naMCI group performed significantly worse than did the NC group, p < 0.05. *p < 0.05; **p < 0.01.

**Figure 5:**
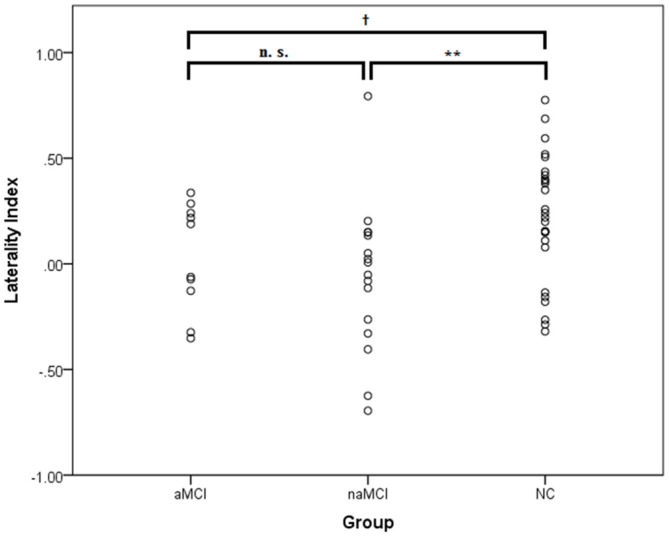
**The laterality index of the [oxy-Hb] changes at the lateral prefrontal regions in the amnestic (aMCI; *n* = 10) and non-amnestic MCI (naMCI; *n* = 16) and NC (*n* = 26) groups.** A positive value implies a lateralization to the left, whereas a negative value implies a lateralization to the right. No outliers (i.e., 2 SDs above or below the group mean) were identified. ^†^*p* < 0.10; **p < 0.01.

For the laterality index (see Figure 5), one-sample *t*-tests showed that none of the MCI groups exhibited a significant frontal lateralization [aMCI: *t*_(9)_ = 0.10, *p* = 0.92, *d* = 0.13; naMCI: *t*_(15)_ = 0.74, *p* = 0.47, *d* = 0.18]. ANOVA showed a significant group difference in the laterality index, *F*_(2,49)_ = 4.36, *p* = 0.018, *η*_p_ = 0.15. It was found that compared to the NC group, the naMCI group had a significantly lower laterality index, *t*_(40)_ = 2.76, *p* = 0.009, *d* = 0.87. A tendency for a lower laterality index was also found for the aMCI group, *t*_(34)_ = 1.72, *p* = 0.095, *d* = 0.59. The two MCI groups did not significantly differ, *t*_(24)_ = 0.76, *p* = 0.45, *d* = 0.31. In addition, all groups exhibited significant [oxy-Hb] increases in the left and right prefrontal regions, *p*s < 0.015. No group differences were found, *p*s > 0.30. None of the MCI groups had significantly greater left than right [oxy-Hb] increases, *p*s > 0.32. In sum, the results suggest that the aMCI and naMCI groups exhibited a similar alteration of frontal activation patterns (e.g., lack of lateralization) during the category fluency task. However, the differences in behavioral performance and neural processing between the MCI and NC groups seem to be driven by the non-amnestic rather than the amnestic subgroups.

## Discussion

In the present study, we examined neural processing during a category fluency task in individuals with MCI by using NIRS. As expected, the behavioral results showed that compared to NC, these individuals generated fewer unique words, made fewer switches between animal subcategories, and generated fewer unique subcategories. In addition, the NIRS results showed that whereas the NC group exhibited a clear left lateralization of frontal activations during the category fluency task, the MCI group did not. A significant and large positive relationship between the category fluency performance and the extent of lateralization of frontal activations was also found. These findings suggest that a reduced lateralization of frontal activations seems to underlie the category fluency deficits in individuals with MCI, especially in those with the non-amnestic subtype.

Our behavioral findings are consistent with previous findings of word generation deficits in individuals with MCI (Nutter-Upham et al., 2008; Brandt and Manning, 2009; Murphy et al., 2006; Libon et al., 2010; Wang et al., 2011; Price et al., 2012; Bertola et al., 2014; Rinehardt et al., 2014; Vogel et al., 2014; Weakley et al., 2013), suggesting that impaired retrieval of semantic knowledge may already be observed at the earliest stage of transition between normal aging and dementia. Moreover, we found that individuals with MCI generated fewer animal subcategories and make fewer switches between subcategories. This is also in line with the literature that impairment in flexible thinking or searching and accessing novel semantic knowledge may underlie the category fluency deficits in individuals with MCI (Raoux et al., 2008; Price et al., 2012; Weakley et al., 2013; Bertola et al., 2014). On the other hand, individuals with MCI made semantic clusters of comparable size. This finding is generally consistent with those reported by previous studies (Raoux et al., 2008; Weakley et al., 2013; Bertola et al., 2014; but see Price et al., 2012). We also found that processing speed does not seem to be a confounding factor of the category fluency deficits in MCI, which is in line with a previous study (Price et al., 2012). In sum, our findings are consistent with the literature and confirmed the presence of a category fluency deficit that seems to be attributed to the impairment in flexible thinking and strategic retrieval of semantic knowledge in MCI.

We found that the NC group demonstrated significant [oxy-Hb] increases in the left and right prefrontal regions, which is consistent with previous findings (Schecklmann et al., 2007; Tupak et al., 2012; Dan et al., 2013; Heinzel et al., 2013, 2015; Liu et al., 2014; Marumo et al., 2014; Holper et al., 2015). In addition, our findings of left-lateralized [oxy-Hb] increases in the NC group are also in line with many of the previous NIRS studies (Fallgatter et al., 1997; Tupak et al., 2012; Dan et al., 2013; Heinzel et al., 2013, 2015), including those that studied older adults (Heinzel et al., 2013, 2015). Our findings of left-lateralized frontal activations during the category fluency task are also consistent with those obtained using other neuroimaging techniques, such as PET (Gourovitch et al., 2000), fMRI (e.g., Weiss et al., 2003; Birn et al., 2010; Wagner et al., 2014), and fTDC (Gutierrez-Sigut et al., 2015). Indeed, the prefrontal cortices, such as the left ventrolateral and dorsolateral prefrontal cortex, have been shown to mediate the strategic retrieval of semantic information (Badre and Wagner, 2007) and flexible thinking (Stuss and Knight, 2013; Yeung et al., 2016) that are demanded by the category fluency task. The frontal activations may reflect the control functions exerted by the frontal lobes during the task performance (Thompson-Schill et al., 1997), and the left lateralization may reflect the verbal nature of the category fluency task (Hellige, 1993). Our findings of correlations between lateralization and the category fluency and naming performance supported the role of lateralization in verbal functioning.

Whereas the NC individuals exhibited a clear pattern of left-lateralized frontal activations, those with MCI in our study did not reveal the same pattern. To our knowledge, the neural processing of individuals with MCI during the category fluency task has not been empirically studied. The present study may serve as a pilot study in this area. Moreover, the present NIRS findings are consistent with previous MRI (Pa et al., 2009; Wang et al., 2009; Chang et al., 2010; Ahn et al., 2011; Zhao et al., 2015) and fMRI studies (e.g., Heun et al., 2007; Kaufmann et al., 2008; Alichniewicz et al., 2012; Papma et al., 2013; Van Dam et al., 2013) that found abnormalities in the frontal lobes of individuals with MCI. In addition, some studies found an association between category fluency performance and cortical atrophy of the (left) prefrontal cortex in individuals with MCI (Ahn et al., 2011; Eastman et al., 2013; Clark et al., 2014). Our findings extend the previous literature by showing a relationship between the category fluency performance and on-task prefrontal dynamics. Accordingly, the cortical atrophy and declined functioning of the prefrontal cortex may lead to a change in lateralization during the category fluency task in individuals with MCI. It should be noted that the absence of frontal lateralization during category fluency or other language tasks could been observed in individuals with early left brain injury (Raja Beharelle et al., 2010) or mental disorders and neurological diseases that exhibited category fluency or language deficits, such as Alzheimer’s dementia (Fallgatter et al., 1997), schizophrenia (Sommer et al., 2001; Weiss et al., 2004), and developmental dysphasia (de Guibert et al., 2011). Because category fluency is mediated more by the left (frontal) regions (e.g., Wagner et al., 2014), a rightward shift of brain activations in the MCI group may reflect the presence of cortical reorganization in which the contralateral regions (i.e., the right hemisphere) are recruited to serve the function that is declining in the specialized regions (i.e., the left hemisphere). Accordingly, reorganization would not take place if the specialized regions are still well functioning, as in the NC group.

This reorganization account is supported by our strong positive relationship between category fluency performance and the extent of left lateralization, implying a beneficial role of lateralization and a detrimental role of the rightward shift in the task performance. In fact, this finding is quite consistent with the previous findings of an association between poorer category fluency performance and greater right frontal activations or greater left frontal atrophy in older adults with NC (Meinzer et al., 2009) and those with MCI (Eastman et al., 2013; Clark et al., 2014). Because the mean [oxy-Hb] changes on each side did not significantly correlate with the task performance although they were in the expected direction (left: *r* = 0.23; right: *r* = −0.04), our study suggests that the relative left-right difference (i.e., the laterality index, *r* = 0.51) may play a more important role than the absolute activation level in determining the category fluency performance. In addition, this brain-behavior relationship was found for both the NC and MCI groups, suggesting that individuals with NC and MCI may lie on the same continuum between performance and lateralization, but those with MCI are situated at a lower end of this continuum. Further studies are needed to clarify whether this same relationship might also be found in demented individuals and that demented individuals would show an even more bilateral or right-lateralized activation pattern than did those with MCI.

We further separated the MCI group into the aMCI and naMCI subtypes given that they were associated with different etiologies (Chao et al., 2009; Pa et al., 2009) and dementia outcomes (e.g., Yaffe et al., 2006). That is, the amnestic subtype is more likely to progress to Alzheimer’s dementia, while the non-amnestic subtype is more likely to progress to other dementia types (Yaffe et al., 2006). We found that although the two MCI groups did not significantly differ in the category fluency impairment, this impairment was more pronounced in the naMCI group compared to the aMCI group. Our finding is consistent with some previous reports of slightly better category fluency performance in (single-domain) aMCI compared to non-amnestic or dysexecutive MCI (Brandt and Manning, 2009; Libon et al., 2010; Vogel et al., 2014). However, our finding is not consistent with other findings of slightly better category fluency performance in naMCI than aMCI (Weakley et al., 2013; Rinehardt et al., 2014). This discrepancy of findings is possibly attributed to the different composition of the MCI groups (i.e., proportion of the single- and multiple-domain subtypes) in different studies. For example, multiple-domain aMCI has been consistently found to have slightly greater category fluency impairment than single-domain aMCI (Brandt and Manning, 2009; Weakley et al., 2013; Bertola et al., 2014). The mixture of the single- and multiple-domain subtypes of our MCI sample may partly explain the subtly better category fluency performance in the aMCI group compared to the naMCI group in the present study. Altogether, these findings illustrate that the MCI construct is heterogeneous and further research with a large sample of various MCI subtypes (i.e., single-domain, multiple-domain, amnestic, non-amnestic) is warranted to verify the level of category fluency impairment associated with each MCI subtype.

In addition, we found that although the aMCI and naMCI groups did not significantly differ in the extent of frontal lateralization during the category fluency task (*p* = 0.45), the frontal abnormality was highly significant in the naMCI group (*p* = 0.009) but was only marginally significant in the aMCI group (*p* = 0.095). These findings suggest the presence of more pronounced frontal dysfunction in individuals with naMCI and confirm the heterogeneity of the MCI construct (Petersen et al., 2014). That is, previous studies have found major disturbances in medial temporal lobe structures, such as the hippocampus and entorhinal cortex in aMCI but in other brain regions in naMCI (Chao et al., 2009; Pa et al., 2009). The current findings of frontal dysfunction mainly in the naMCI group are consistent with previous reports of frontal abnormalities that were specific to (dysexecutive) naMCI (Chao et al., 2009; Pa et al., 2009). In addition, although frontal dysfunction is not a major characteristic of patients with aMCI (Pa et al., 2009), the tendency for our aMCI group to also exhibit frontal dysfunction may be attributed to an over-representation of individuals with multiple-domain aMCI that had executive dysfunction (i.e., significantly larger STT-B: time) in addition to memory deficits. Alternatively, it is possible that disturbances in medial temporal lobe structures may alter the normal functioning of the prefrontal cortex during cognitive tasks since the medial temporal lobe is closely connected with the frontal lobe (Simons and Spiers, 2003). Given these speculations and the heterogeneity of the MCI construct, further studies with a large sample of individuals with single-domain aMCI (i.e., pure memory problems) may help explain the observed tendency for frontal dysfunction in our aMCI group.

Our findings have several important implications. First, we found that individuals with MCI did not exhibit a lateralization of frontal activations during the category fluency task. This finding suggests that the lateralization pattern may serve as a neural marker for distinguishing between normal aging and MCI. Developing and identifying objective markers of MCI is important because early detection of patients at the prodromal stage of dementia offers the best potential for intervention (Ernst et al., 1997). Given that NIRS is time- and cost-effective and easy to administer, it seems to be a promising tool for probing the neural functioning in various clinical populations. In addition, our lateralization finding may also serve as a neural marker for the evaluation of the effectiveness of interventions that target the MCI population in the future. Furthermore, our study showed that the NC and MCI were on the same continuum between the category fluency performance and lateralization, suggesting that cognitively normal older adults that exhibited both a low category fluency score and a small extent of lateralization may have increased risks for the development of MCI or dementia. To verify this speculation, longitudinal studies that track cognitive changes in cognitively normal older adults that exhibited a large or a small lateralization are needed.

Although the present study has enhanced our understanding of the neural basis of the category fluency deficits in individuals with MCI, some limitations must be mentioned. First, the NIRS measurement was restricted to the prefrontal regions. Given that the category fluency performance may engage non-frontal regions (Birn et al., 2010; Wagner et al., 2014), the MCI group might have altered activations in non-frontal regions. In addition, we primarily combined the amnestic and non-amnestic MCI subtypes into one single MCI group because of the non-significant differences between the two and the relatively small sample size. Some studies reported that the amnestic subtype was more likely to progress to Alzheimer’s dementia, while the non-amnestic subtype was more likely to progress to other dementia types (e.g., Yaffe et al., 2006), suggesting different etiologies between the two subtypes. Further studies with a larger sample are needed to clarify if the two MCI subtypes would differ in the neural processing during the category fluency task and if the lateralization pattern can reliably predict specific forms of dementia. Furthermore, because structural brain images were not acquired, the relationship between lateralization and brain structure remains to be determined. Moreover, although we demonstrated that education was not a confounding factor of the present findings, education was not closely matched between the groups. Moreover, females were over-represented in the sample. Thus, further investigations with a larger sample that has a more even gender distribution and a closer match on education are warranted.

In conclusion, we found that individuals with MCI had category fluency deficits and that impairment in flexible thinking and strategic search and access of semantic information seem to underlie these deficits. In addition, we extend the literature by providing the neural basis of the category fluency deficits in these individuals, showing that they did not exhibit a left-lateralization of frontal activations, unlike their cognitively normal counterparts, during the category fluency task. This rightward shift of activations may reflect the presence of cortical reorganization, in which the contralateral hemispheric takes over the function that is declining in the specialized hemisphere. This interpretation is supported by the finding of a strong relationship between the lack of lateralization and category fluency deficits. To verify the potentiality of the use of NIRS for clinical diagnosis and the evaluation of the effectiveness of interventions that target the MCI population, further studies are needed. Our findings suggest that a neural disturbance during the category fluency task may already be present at the prodromal stage of dementia. In addition, given that the category fluency impairment and frontal abnormalities were highly significant in the naMCI group but only marginally significant in the aMCI group, our study also highlights the heterogeneity of the MCI construct.

## Author Contributions

ASC, SLS, JW, TK, DHKS, and RY conceived the study. All authors led the design of the experiment. SLS and RY led the field implementation. MKY, SLS, and RY led the data collection. MKY prepared the data for analyses. MKY and ASC engaged in the analyses. MKY, ASC, JW, TK, DHKS, RY contributed to the interpretation of the data. MKY and ASC drafted the first manuscript. All authors work on the revisions of the drafts, critically contributed to its content and approved the final version of the manuscript.

## Conflict of Interest Statement

The authors declare that the research was conducted in the absence of any commercial or financial relationships that could be construed as a potential conflict of interest.
